# The Associations between Sleep Duration and Sleep Quality with Body-Mass Index in a Large Sample of Young Adults

**DOI:** 10.3390/ijerph15040758

**Published:** 2018-04-15

**Authors:** Tomislav Krističević, Lovro Štefan, Goran Sporiš

**Affiliations:** Faculty of Kinesiology, University of Zagreb, 10 000 Zagreb, Croatia; tomislav.kristicevic@kif.hr (T.K.); sporis.79@gmail.com (G.S.)

**Keywords:** university students, sleeping habits, nutritional status, logistic regression analysis

## Abstract

Background: The main aims of this study were to explore the associations between time spent in bed (as a proxy of sleep duration) and sleep quality with overweight/obesity status in a large sample of young adults. Methods: In this cross-sectional study, participants were 2100 university students (49.6% of women). We used Pittsburgh Sleep Quality Index (PSQI) questionnaire to assess time spent in bed and sleep quality. Body-mass index (BMI) was self-reported and dichotomized as normal (<25 kg/m^2^) vs. overweight/obesity (≥25 kg/m^2^) status. Results: In model 1, both short (<6 h/day, OR = 2.72; 95% CI 1.27 to 5.84) and long (>10 h/day, OR = 3.38; 95% CI 2.12 to 5.40) time spent in bed were associated with a greater likelihood of being overweight/obese. In model 2, poor sleep quality (>5 points, OR = 1.45; 95% CI 1.14 to 1.83) was associated with a greater likelihood of being overweight/obese. After entering time spent in bed and sleep quality simultaneously into the model 3, both short (OR = 2.64; 95% CI 1.23 to 5.66) and long (OR = 3.27; 95% CI 2.04 to 5.23) time spent in bed and poor sleep quality (OR = 1.40; 95% CI 1.10 to 1.78) were associated with overweight/obesity status. Conclusions: Our results show that both short and long time spent in bed and poor sleep quality are associated with overweight/obesity status in young adults. Special interventions and policies that use both sleep duration and sleep quality as protective factors against overweight/obesity are warranted.

## 1. Introduction

High body-mass index (BMI) has become one of the biggest public health problems worldwide in the last two decades [1]. It has been well-documented that BMI over 25 kg/m^2^ in the general population represents a risk factor for cardiovascular, metabolic, and musculoskeletal diseases [2,3]. One recent study has shown that the prevalence of overweight/obesity status in children, adolescents, and adults has risen by 10 times over the last forty years, with a special emphasis on high and middle-income countries [4].

Previous studies have shown that sufficient and regular physical activity and balanced nutrition may serve as a prevention methods against overweight/obesity [5,6], although recent meta-analysis showed non-significant effects [7,8]. Recently, great attention has been given to sleep duration [9,10,11,12] and sleep quality [12,13,14,15] associated with BMI. However, results from these studies are inconsistent. While some studies have shown a negative association between sleep duration and BMI [10,11], others have found U-shaped [9] or even no association [16]. Also, inconsistent results have been found for the association between sleep quality and BMI, with some studies showing that poor sleep quality is associated with an increased likelihood of being overweight/obese [13,14], while others reporting no association [15].

The population of young adults is at extreme risk, since it goes through lifestyle changes in terms of extensive electronic media use and academic demands, which can potentially lead to insufficient sleep and poor sleep quality [17], often accompanied with poor diet [18] and lack of physical activity (PA) [19]. According to the aforementioned, there has been a lack of studies investigating simultaneous associations between sleep duration and sleep quality with BMI in young adults.

Thus, the main aims of this study were to explore separate and simultaneous associations between sleep duration and sleep quality with overweight/obesity status in a large sample of young adults.

## 2. Materials and Methods

### 2.1. Participants

We conducted a survey among university students in Zagreb, the capital city of Croatia, with approximately 1,000,000 citizens. The University of Zagreb is composed of 33 faculties (departments) and between 65,000 and 70,000 students attend the University every year. A random sampling approach was used to select faculties. At the first stage, we randomly selected 8 out of 33 faculties. The randomization was done with replacement, in which each faculty had unique number and was drawn from the box. At the second stage, we contacted teachers from each faculty to help us organize the sampling procedure. A recruitment announcement was sent via emails and e-newsletter to the teachers with a request to pass the study information to students. All 8 faculties agreed to take part in the study, representing 2320 students enrolled in the 2017 academic year. Of these, 2100 students (1041 men and 1059 women, aged 18–24 years) provided full data (90.5%) and were enrolled in further analysis. Students came from a variety of social (psychology, political sciences, economy and business), technical (computing, information technologies, electrical engineering, civil engineering, mechanical engineering, graphics arts, and naval architecture) and health-related (medical doctors, physiotherapists, nurses) sciences. Before the main analysis, we examined the differences between the participants and non-participants in terms of height, weight, BMI, and sleeping patterns. No significant differences were observed and no potential bias was made (*p* = 0.21–0.74). All the analysis and procedures were anonymous and in accordance with the Declaration of Helsinki and approved by the Institutional Review Board of the Faculty of Kinesiology (ethics code number: 16/2017).

### 2.2. Body-Mass Index

Participants self-reported their height in meters (m) and weight in kilograms (kg), from which body-mass index (BMI, kg/m^2^) was calculated. As proposed by one previous study [10], before the study began, we had chosen 35 men and 40 women to validate self-reported height and weight with the objective measure taken by trained survey staff. Pearson’s coefficient of correlation showed excellent relationship between two measures in men (r = 0.96) and women (r = 0.97). For the purpose of this study, we dichotomized the outcome, in which participants with the value <25 kg/m^2^ collapsed into normal vs. those ≥25 kg/m^2^ with overweight/obesity status [15]. Although not appropriate as a clinical tool, self-reported BMI serves as a valid tool for epidemiological surveys, especially in young adults [20].

### 2.3. Time Spent in Bed

To assess time spent in bed, we asked the following questions: “What time do you usually go to bed during weekday?” and “What time do you usually get up during weekday?” The same two questions were assessed for weekend days [10]. These questions have been taken from previously validated questionnaires [21].

Time spent in bed was calculated by subtracting “go to bed” time from “get up” time. Average weekly value of sleep duration was calculated using following equation:

Average time spent in bed = [(week day time spent in bed × 5) + (weekend day time spent in bed × 2)]/7(1)

For the purpose of this study, we divided the numerical values of time spent in bed into 5 categories: (1) <6 h, (2) 6–7 h, (3) 7.1–9 h, (4) 9.1–10 h, and (5) >10 h of time spent in bed. Our referent value was 7-9 h of sleep, since sleeping less or more than recommended 7–9 h has negative health consequences, such as weight gain and higher risk of cardiovascular, metabolic, and musculoskeletal disorders [22]. Previous studies have shown moderate agreement between self-reported and objectively measured (actigraphy) sleep duration [23], and this tool has often been used to assess sleep duration in young adults [10,12].

### 2.4. Sleep Quality

To assess sleeping habits, we used Pittsburgh Sleep Quality Questionnaire (PSQI), highly reliable and valid instrument specifically designed to measure sleep quality [24]. It is composed of 19 questions, which create 7 major components. Each component is scored from 0 to 3 points, in which lower point denotes no problems, while higher score denotes worsening problems in following order: (1) subjective sleep quality (very good vs. very bad), (2) sleep latency (≤15 min to >60 min), (3) sleep duration (≥7 h to <5 h), (4) sleep efficiency (≥85% to <65% h sleep/hours in bed), (5) sleep disturbances (not during the past month to ≥3 times per week), (6) use of sleeping medications (none to ≥3 times a week) and (7) daytime dysfunction (not a problem to a very big problem) [24]. All 7 components are then summed up to create a scale from 0 to 21 points. For the purpose of the present study, we dichotomized the result into two categories: (1) ≤5 (good sleep quality) vs. (2) >5 (poor sleep quality), as proposed by Buysse et al. [24] The reliability of the PSQI in our study was satisfactory (Cronbach’s α = 0.73).

### 2.5. Covariates

Self-rated health was assessed using one-item question: “In general, how would you rate your health?” Responses were arranged along a five item Likert-type scale as follows: (1) very poor, (2) poor, (3) fair, (4) good, and (5) excellent. We dichotomized the responses as “poor health“(responses very poor and poor) vs. “good health“(responses fair, good, and excellent). This measure has previously been shown as a valid predictor of mortality and has often been used in a sample of young adults [25]. Socioeconomic status was assessed with one-item question: “How would you perceive your socioeconomic status, according to your parents’ occupational status?” Responses were arranged according to 3 item answers as follows: (1) below average, (2) average, and (3) above average. Smoking status was categorized as: (1) non-smoker, (2) former smoker, and (3) present smoker. Binge drinking was assessed by one-item question: “How often do you have (for men) 5 or more and (for women) 4 or more drinks on one occasion?” [26]. Those who had (for men) 5 or more and (for women) 4 or more drinks on one occasion were categorized as “Yes” compared to “No” group, which had fewer drinks on one occasion. The presence or absence of a chronic disease was asked by one-item question: “Have you ever been told by a doctor that you suffer from any kind of chronic disease?” with “Yes” and “No” answers. Psychological distress was assessed by using Kessler’s 6-item questionnaire: (1) “How often during the past 30 days did you feel nervous?”, (2) “How often during the past 30 days did you feel hopeless?”, (3) “How often during the past 30 days did you feel restless or fidgety?”, (4) “How often during the past 30 days did you feel so depressed that nothing could cheer you up?”, (5) “How often during the past 30 days did you feel that everything was an effort?”, and (6) “How often during the past 30 days did you feel worthless?” [27]. Each question was scored from 0 (none of the time) to 4 (all of the time). Scores of each question were summed up between 0 and 24, with lower score indicating lower level of psychological distress. Kessler et al. [27] showed that responses <13 points vs. ≥13 points discriminated participants without and with psychological distress. To assess physical activity in the last 7 days, we used International Physical Activity questionnaire, a reliable and valid instrument designed to measure physical activity in respondents between ages 18–65 [28]. We created a dichotomized variable, in which “sufficiently active” participants took part in at least (1) 150 min/week in moderate physical activity, or (2) 75 min of vigorous physical activity, or (3) an equivalent combination of both vs. “insufficiently active” participants [29].

### 2.6. Data Analysis

Basic descriptive statistics of the study participants are presented as frequencies (N) and percentages (%). Differences between categorical variables were analyzed using Chi-square test. To examine the associations between time spent in bed and sleep quality with overweight/obesity status, we used multiple logistic regression analysis. We calculated odds ratios (ORs) with 95% confidence intervals (95% CIs), according to levels of time spent in bed and sleep quality. First, we examined the association between time spent in bed and overweight/obesity status in Model 1. Next, we examined the association between sleep quality and overweight/obesity status in Model 2. Finally, we entered simultaneously time spent in bed and sleep quality into the Model 3. All models were additionally adjusted for gender, self-rated health, socioeconomic status, smoking status, alcohol consumption, the presence or absence of chronic diseases, psychological distress, and physical activity. The interaction effects between time spent in bed (*p* = 0.24) and sleep quality (*p* = 0.06) with gender were not statistically significant, so we dropped the gender-stratified analysis. Also, we calculated the differences between students studying for different professions and found no significant differences in terms of BMI (*p* = 0.235), time spent in bed (0.127), and sleep quality (*p* = 0.468). Significance was set up at α ≤ 0.05, and it was two sided (2-sided). All the analysis were performed in Statistical Package for Social Sciences Software, ver. 22 (IBM Corp., Armonk, NY, USA).

## 3. Results

Basic descriptive statistics of the study participants are presented in Table 1. Approximately 18.0% of participants with normal BMI status spent less time in bed (<7 h), and 3.0% of them spent more time in bed (>10 h). On the other hand, more than 20.0% of participants with overweight/obesity status were classified as those spending a short amount of time in bed, and almost 10.0% of them spent more time in bed. Higher percentage of participants with BMI ≥25 kg/m^2^ had poor sleep quality (≈45.0%) compared with participants with BMI <25 kg/m^2^ (36.0%). Interestingly, higher percentage of participants with normal BMI reported having good self-rated health (≈94.0%), and lower percentage of them reported having a chronic disease (91.7% vs. 86.5%, *p* = 0.002).

Table 2 shows the associations between time spent in bed and sleep quality with overweight/obesity status in the study participants. When only time spent in bed with potential covariates was entered into the model (Model 1), very short (OR = 2.72; 95% CI 1.27 to 5.84) and very long (OR = 3.38; 95% CI 2.12 to 5.40) time spent in bed were associated with higher likelihood of being overweight/obese. When only sleep quality with potential covariates was entered into the model (Model 2), poor sleep quality was associated (OR = 1.45; 95% CI 1.14 to 1.83) with higher likelihood of being overweight/obese. Finally, when both time spent in bed and sleep quality were entered simultaneously into the model and adjusted for potential covariates, very short (OR = 2.64; 95% CI 1.23 to 5.66) and very long (OR = 3.27; 95% CI 2.04 to 5.23) time spent in bed and poor sleep quality (OR = 1.40; 95% CI 1.10 to 1.78) were all associated with higher likelihood of being overweight/obese.

## 4. Discussion

The main aims of the present study were to explore separate and simultaneous associations between sleep duration and sleep quality with overweight/obesity status in a large sample of young adults. Our results showed that both short and long time spent in bed and poor sleep quality were associated with having overweight/obesity status.

Previous findings from epidemiological studies conducted on a sample of young adults have been very inconsistent. While some of them reported inverse association between sleep duration and BMI [10], others reported positive association only between short sleep duration and BMI [12] and no association between long sleep duration and BMI [12]. In general, findings consistently show the association between short sleep duration and BMI [10,12]. Short sleep duration is associated with many hormonal changes, especially with decreased levels of leptin (hormone that suppresses appetite) and increased levels of ghrelin (hormone that increases appetite), which potentially mediate the association between short sleep and BMI [30]. Moreover, a study by Markwald et al. [31] showed that 5 consistent days of insufficient sleep increased energy needs and energy intake and decreased responsivity to gut fullness and satiety hormones. From a physiological point of view, energy needs are higher during sleep loss, which potentially leads to a decreased level of leptin and increased level of ghrelin, and thus to excessive weight gain [31]. Another potential factor influencing increased levels of BMI is circadian rhythms [32]. In adolescent population, it has been shown that going to bed early has beneficial effects on BMI, while those reporting going to bed late are 1.47 more likely to be overweight/obese or 2.16 times more likely to be obese [33]. The association between long sleep duration and elevated levels of BMI is still unclear. Previous studies conducted on adolescents have shown that long sleepers have greater likelihood for being overweight/obese [9,34]. It has been reported that long sleepers are less physically active, which can potentially reduce energy expenditure [35]. However, even when we adjusted for physical activity, long sleep duration remained associated with increased likelihood for being overweight or obese.

Next, our results showed that poor sleep quality was associated with increased likelihood of being overweight/obese. One recent meta-analysis showed that poor sleep duration was associated with overweight/obesity status in children, adolescents, and young adults, independent of sleep duration [13]. Another study showed no association between sleep quality and BMI [15]. However, it should be highlighted that the aforementioned group of authors had a convenient and relatively small sample size (N = 515), which possibly influenced the power of the study. A study by Hung et al. [14], which was conducted on a large Chinese population sample (N = 2803), showed that those participants who were overweight/obese had 40.0% and 60.0% higher risk of being poor sleepers. As for the association between long sleep and BMI, the mechanism underlying the association between sleep quality and BMI is still unclear [14]. It has been documented that poor sleep-quality changes appetite regulation mechanism, possibly leading to poor food choice and increased caloric intake [36,37].

This study has several limitations. First, this study was cross-sectional; we cannot exclude the possibility of reverse causality, that is, higher levels of BMI led to poorer sleep quality and both short and long time spent in bed. However, we did adjust for numerous potential covariates affecting both BMI, time spent in bed, and sleep quality. Second, we used self-reported data to assess BMI, time spent in bed, and sleep quality. Although subjective, we presented very good correlation between self-reported and measured BMI. Also, as highlighted by one recent study, PSQI questionnaire represents a valid and reliable instrument for assessing sleep quality [13]. Third, we used time spent in bed as a proxy of sleep duration, which does not reflect true sleep duration in the study participants. As a recommendation, direct question of sleep duration (“How long do you sleep during the night?”) should be assessed in the future studies. Fourth, although adjusted for numerous covariates, we did not adjust for dietary intake. Previous studies have shown that both sleep quality and sleep duration are associated with diet [36]. Fifth, we also did not adjust for sleep apnoea. Previous studies have shown that sleep apnoea is associated with BMI [38,39]. Specifically, Busetto et al. [39] reported that a 15% reduction in baseline body weight substantially increased the mean pharyngeal cross-sectional area of the throat and significantly decreased the severity of obstructive sleep apnoea. In a study by Wall et al. [38], participants with a BMI recorded >40 kg/m^2^ were more than 27 times more likely to have obstructive sleep apnoea. Thus, along with dietary intake, future studies should also use sleep apnoea as a covariate. Finally, the outcome variable was only classified in participants with normal (<25 kg/m^2^) vs. overweight/obese (≥25 kg/m^2^) status. We grouped the underweight participants under the participants with normal BMI and our findings must be taken with caution. However, such categorization has been done in previous studies [10,15].

## 5. Conclusions

The results of the present study show separate and simultaneous associations between time spent in bed and sleep quality with overweight/obesity status. Specifically, both short and long time spent in bed and poor sleep quality are associated with higher likelihood of having overweight/obese status in a large sample of young adults. From the clinical point of view and based on our findings, physicians should not use only time spent in bed (as a proxy of sleep duration); rather, they should use sleep duration combined with sleep quality to achieve a better understanding of the association between sleeping habits and BMI. Moreover, future studies using objective and valid measures should track sleeping habits and overweight/obesity status for a longer period of time, in order to establish causal associations and help to determine whether sleep duration and sleep quality have a protective role against being overweight/obese.

## Figures and Tables

**Table 1:** Basic descriptive statistics of the study participants, Croatia (2017).

Study Variables	<25 kg/m^2^ (N = 1706)	≥25 kg/m^2^ (N = 394)	*p*-Value *
	N (%)	N (%)	
Time spent in bed			
<6 h	20 (1.2)	13 (3.3)	
6–7 h	280 (16.4)	67 (17.0)	
7.1–9 h	1142 (66.9)	218 (55.3)	
9.1–10 h	210 (12.3)	57 (14.5)	
>10 h	54 (3.2)	39 (9.9)	<0.001
Sleep quality			
Good	1092 (64.0)	218 (55.3)	
Poor	614 (36.0)	176 (44.7)	0.002
Gender			
Men	769 (45.1)	272 (69.0)	
Women	937 (54.9)	122 (31.0)	<0.001
Self-rated health			
Good	1598 (93.7)	337 (85.5)	
Poor	108 (6.3)	57 (14.5)	<0.001
Socioeconomic status			
High	245 (14.4)	59 (15.0)	
Medium	1428 (83.7)	316 (80.2)	
Poor	33 (1.9)	19 (4.8)	0.003
Smoking status			
No	1213 (71.1)	266 (67.5)	
Former	88 (5.2)	27 (6.9)	
Yes	405 (23.7)	101 (25.6)	0.25
Binge drinking			
No	1248 (73.2)	282 (71.6)	
Yes	458 (26.8)	112 (28.4)	0.53
Chronic diseases			
No	1564 (91.7)	341 (86.5)	
Yes	142 (8.3)	53 (13.5)	0.002
Psychological distress			
Low	1524 (89.3)	354 (89.8)	
High	182 (10.7)	40 (10.2)	0.86
Physical activity			
Sufficiently active	1322 (77.5)	304 (77.2)	
Insufficiently active	384 (22.5)	90 (22.8)	0.89

* Chi-square test.

**Table 2:** Separate and simultaneous associations between time spent in bed and sleep quality with overweight/obesity status of the study participants, Croatia (2017).

Study Variables	Model 1	Model 2	Model 3
	OR (95% CI)	OR (95% CI)	OR (95% CI)
Time spent in bed			
<6 h	2.72 (1.27 to 5.84) **		2.64 (1.23 to 5.66) *
6–7 h	1.20 (0.87 to 1.64)		1.15 (0.84 to 1.58)
7.1–9 h	Ref.		Ref.
9.1–10 h	1.29 (0.92 to 1.80)		1.27 (0.91 to 1.79)
>10 h	3.38 (2.12 to 5.40) ***		3.27 (2.04 to 5.23) ***
Sleep quality			
Good		Ref.	Ref.
Poor		1.45 (1.14 to 1.83) **	1.40 (1.10 to 1.78) **
Gender			
Men	Ref.	Ref.	Ref.
Women	0.31 (0.24 to 0.40) ***	0.31 (0.24 to 0.40) ***	0.31 (0.24 to 0.39) ***
Self-rated health			
Good	Ref.	Ref.	Ref.
Poor	2.51 (1.70 to 3.72) ***	2.83 (1.93 to 4.14) ***	2.46 (1.66 to 3.64) ***
Socioeconomic status			
High	Ref.	Ref.	Ref.
Medium	1.11 (0.81 to 1.54)	1.05 (0.76 to 1.44)	1.12 (0.81 to 1.54)
Poor	2.08 (1.06 to 4.07) *	1.93 (1.00 to 3.74) *	2.06 (1.05 to 4.04) *
Smoking status			
No	Ref.	Ref.	Ref.
Former	1.09 (0.82 to 1.44)	1.13 (0.86 to 1.49)	1.08 (0.82 to1.44)
Yes	1.30 (0.80 to 2.10)	1.38 (0.86 to 2.23)	1.27 (0.79 to 2.06)
Binge drinking			
No	Ref.	Ref.	Ref.
Yes	1.19 (0.92 to 1.55)	1.21 (0.93 to 1.56)	1.20 (0.92 to 1.57)
Chronic diseases			
No	Ref.	Ref.	Ref.
Yes	1.45 (1.01 to 2.09) *	1.54 (1.07 to 2.20) *	1.43 (1.00 to 2.06) *
Psychological distress			
<13 points	Ref.	Ref.	Ref.
≥ 13 points	0.97 (0.66 to 1.43)	0.86 (0.58 to 1.27)	0.88 (0.59 to 1.31)
Physical activity			
Sufficiently active	Ref.	Ref.	Ref.
Insufficiently active	1.08 (0.81 to 1.45)	1.07 (0.80 to 1.42)	1.04 (0.78 to 1.40)

Model 1 examines the association between time spent in bed and overweight/obesity status adjusted for gender, self-rated health, socioeconomic status, smoking status, binge drinking, chronic diseases, psychological distress, and physical activity. Model 2 examines the association between sleep quality and overweight/obesity status adjusted for gender, self-rated health, socioeconomic status, smoking status, binge drinking, chronic disease, psychological distress, and physical activity. Model 3 examines the associations of time spent in bed and sleep quality entered simultaneously into the model with overweight/obesity status adjusted for gender, self-rated health, socioeconomic status, smoking status, binge drinking, chronic diseases, psychological distress, and physical activity. *** *p* < 0.001, ** *p* < 0.01, * *p* < 0.05.

## Abbreviations

BMI	Body-mass index
Kg	kilograms
M	meters
OR	odds ratio
PA	physical activity
PSQI	Pittsburgh Sleep Quality Index
95% CI	95 percent confidence interval
